# Draft genome sequences of *Lactobacillus mulieris* strains isolated from the female urinary tract

**DOI:** 10.1128/mra.01121-23

**Published:** 2023-12-22

**Authors:** Natalie Stegman, Maria Steiling, Cerena Sedano, Briana Jackson, Catherine Putonti

**Affiliations:** 1 Bioinformatics Program, Loyola University Chicago, Chicago, Illinois, USA; 2 Department of Biology, Loyola University Chicago, Chicago, Illinois, USA; Rochester Institute of Technology, Rochester, New York, USA

**Keywords:** *Lactobacillus mulieris*, urinary tract

## Abstract

*Lactobacillus mulieris* is a frequent member of the healthy female urogenital tract. Here, we present the draft genomes of two *L. mulieris* strains isolated from urine samples: UMB0446 and UMB3420. The draft genomes presented here further expand our understanding of the female urinary microbiome.

## ANNOUNCEMENT

The healthy urogenital microbiota commonly harbors *Lactobacillus* species (1, 2). The species *Lactobacillus mulieris* was described in March 2020 from a urine isolate (3), leading to the subsequent reclassification of numerous *Lactobacillus jensenii* strains based on average nucleotide identity (ANI) (3, 4). *L. jensenii* and *L. mulieris* are members of both the urinary and vaginal microbiota (4–6). More recently*, L. mulieris* abundance in the vaginal microbiota has been associated with a decreased risk of spontaneous preterm birth (5).

In an effort to further characterize lactobacilli from the urinary tract, two strains were sequenced. Strain UMB0446 was obtained via transurethral catheterization of a female without lower urinary tract symptoms (Loyola University Chicago, IRB# 207102), and strain UMB3420 was obtained from a voided urine from a female with Type 2 diabetes (Loyola University Chicago, IRB# 204197) (7). Urine samples were cultured using the enhanced quantitative urine culture method (8) and identified as *L. jensenii* via matrix-assisted laser desorption/ionization time-of-flight (MALDI-TOF) (9) prior to storage at −80°C. Each freezer stock was streaked on deMan-Rogosa-Sharpe (MRS) agar plates and incubated under 5% CO_2_ and 35°C for 48 hours. Then, the MRS medium supplemented with 1  mL/L of Tween 80 (Sigma-Aldrich) was inoculated with a single colony and incubated as described above. DNA was extracted from the liquid culture using Qiagen’s DNeasy blood and tissue kit following the manufacturer’s protocol for Gram-positive bacteria. DNA was quantified using a Qubit fluorometer.

DNA library preparation and sequencing were conducted by SeqCenter (Pittsburgh, PA USA). Libraries were prepared using the tagmentation-based and PCR-based Illumina DNA Prep kit and custom IDT 10 bp unique dual indices with a target insert size of 320 bp and sequenced on the Illumina NovaSeq 6000 platform, producing 2 × 151 bp paired-end reads. Raw reads were trimmed using BBDuk (part of the BBMap suite, v.39.01; sourceforge.net/projects/bbmap/) with the following parameters: qtrim = rl, ftl = 15, ftr = 135, maq = 20, maxns = 0, and trimq = 20. The filtered reads were assembled using SPAdes v.5.5 with the --only-assembler option (10). Genome coverage was calculated using the BBWrap script in BBMap. Genome assemblies were annotated via Prokaryotic Genome Annotation Pipeline v.6.6 (11). Genome sequencing and assembly statistics are listed in Table 1.

**TABLE 1:** Genome sequencing and assembly statistics

Strain	SRA accession no.	No. of raw reads	Assembly accession no.	Genome length (bp)	No. of contigs	Contig N50 (bp)	GC content (%)	Coverage
UMB0446	SRR26087528	2,966,278	GCA_032376715.1	1,680,350	49	65,916	34.20	256.356 x
UMB3420	SRR26087530	2,935,524	GCA_032376585.1	1,821,790	44	84,248	34.10	235.048 x

As both strains were initially identified as *L. jensenii* via MALDI-TOF, performed prior to the description of *L. mulieris*, we first identified the species of the two strains via ANI. Each assembly was compared to *L. mulieris* c10Ua161M^T^ and *L. jensenii* ATCC 25258^T^ using JSpeciesWS (ANIb method) (12), confirming that both strains are members of this *L. mulieris*. ANI values were 96.30 for UMB0446 and 96.00 for UMB3420. Both genome assemblies were screened for biosynthetic gene clusters via antiSmash v.7.0.1 with default parameters (13). UMB0446 encodes for a ribosomally synthesized and post-translationally modified peptide, or RIPP-like cluster, which is characteristic of a putative subspecies of *L. mulieris* (14). UMB3420 encodes for an non-ribosomal peptide synthetase cluster; this is uncharacteristic of *L. mulieris* genomes and is rather characteristic of *L. jensenii* (14). These findings further our collection of the genetic diversity of this species in the female urogenital tract.

## Data Availability

Raw reads and draft assemblies have been deposited in GenBank. Table 1 lists the Sequence Read Archive accession numbers and genome assembly accession numbers.
